# Enhanced Brain Responses to Pain-Related Words in Chronic Back Pain Patients and Their Modulation by Current Pain

**DOI:** 10.3390/healthcare4030054

**Published:** 2016-08-10

**Authors:** Alexander Ritter, Marcel Franz, Christian Puta, Caroline Dietrich, Wolfgang H. R. Miltner, Thomas Weiss

**Affiliations:** 1 Department of Biological and Clinical Psychology, Friedrich Schiller University of Jena, Am Steiger 3, Haus 1, Jena D-07743, Germany; alexander.ritter@uni-jena.de (A.R.); marcel.franz@uni-jena.de (M.F.); caroline.dietrich@uni-jena.de (C.D.); wolfgang.miltner@uni-jena.de (W.H.R.M.); 2 Section of Neurological Rehabilitation, Hans–Berger Department of Neurology at Jena University Hospital, Erlanger Allee 101, Jena D-07747, Germany; 3 Department of Sports Medicine and Health Promotion, Friedrich Schiller University of Jena, Wöllnitzer Str. 42, Jena D-07749, Germany; christian.puta@uni-jena.de

**Keywords:** chronic back pain, semantic processing, current pain, fMRI

## Abstract

Previous functional magnetic resonance imaging (fMRI) studies in healthy controls (HC) and pain-free migraine patients found activations to pain-related words in brain regions known to be activated while subjects experience pain. The aim of the present study was to identify neural activations induced by pain-related words in a sample of chronic back pain (CBP) patients experiencing current chronic pain compared to HC. In particular, we were interested in how current pain influences brain activations induced by pain-related adjectives. Subjects viewed pain-related, negative, positive, and neutral words; subjects were asked to generate mental images related to these words during fMRI scanning. Brain activation was compared between CBP patients and HC in response to the different word categories and examined in relation to current pain in CBP patients. Pain-related words vs. neutral words activated a network of brain regions including cingulate cortex and insula in subjects and patients. There was stronger activation in medial and dorsolateral prefrontal cortex (DLPFC) and anterior midcingulate cortex in CPB patients than in HC. The magnitude of activation for pain-related vs. negative words showed a negative linear relationship to CBP patients’ current pain. Our findings confirm earlier observations showing that pain-related words activate brain networks similar to noxious stimulation. Importantly, CBP patients show even stronger activation of these structures while merely processing pain-related words. Current pain directly influences on this activation.

## 1. Introduction 

Processing and perceptual evaluation of noxious events and their underlying neural substrates are strongly modulated by psychological variables such as attention [1,2,3,4], emotion [5,6,7,8,9,10], expectation [11,12], and learning [13,14]. Furthermore, several studies indicate that environmental semantic and visual pain-related cues can induce activity in structures of the brain that process, among others, nociceptive information [15,16,17] even when no noxious stimulus is applied [18,19]. Based on Hebb’s concept of cell assemblies, it can be assumed that whenever we experience pain, its semantic and emotional representations are activated simultaneously with neural structures that process noxious events and constitute the experience of pain [20,21]. Consequently, words that are used to describe pain-related experiences were found to alter pain itself [22,23], and to activate brain structures engaged in the processing of noxious stimuli, e.g., anterior cingulate cortex, insula, secondary somatosensory cortex (SII), prefrontal cortex, and parietal cortex [24,25].

It has been shown that chronic back pain (CBP) patients differ from healthy controls (HC) in structural [26], functional [27,28], and neurochemical brain parameters [29,30]. In CBP patients, pain is a common everyday experience and pain has been expressed hundreds of times throughout the course of chronic pain development. It was suggested that CBP patients as chronic pain sufferers should have developed a strong pain network and a strong link of this network to their lexicon of pain terms. Accordingly, chronic pain patients exhibit larger event-related potentials (ERPs) to painful stimuli [31], larger late ERP amplitudes when noxious stimuli were primed by pain-related words [14], and stronger blood oxygenation level dependent (BOLD) responses even when pain was not attended [32,33,34]. Furthermore, chronic pain patients showed larger late ERP magnitudes in response to pain-related words and rated such words more negatively than neutral words, indicating that pain-related words draw similar attention and information processing as if an actually painful stimulus would have been processed [35]. In summary, the processing of pain-related words leads to enhanced behavioral and neuronal responses even when semantic processing is precluded from conscious access.

The present study aimed to extend previous findings by investigating the processing of pain-related words in CBP patients with actually ongoing (current) back pain. It was expected that the presence of current pain would activate the brain regions important for the analysis of pain and, thereby, enhance the processing of pain-related words. These assumptions lead to the following hypotheses: H1—CBP patients exhibit different valence, arousal, and pain relevance to pain-related words compared to HC; H2—CBP patients vs. HC show a stronger activation during the processing of pain-related than non-pain-related word categories and to negative words; and H3—There is a linear relationship between the strength of current pain and the measured BOLD response during the processing of pain-related words in CBP patients.

## 2. Materials and Methods

### 2.1. Patients and Controls

Participants of this study were recruited by advertisement at the university or by personal contact. Thirteen patients with CBP (2 men; 23–56 years old, mean age = 44.3 years) and thirteen pain-free HC (2 men; 24–58 years old, mean age = 46.5 years), matched for gender, age, and education, participated in this study as paid volunteers. Sociodemographic and clinical characteristics of participants are summarized in Table 1. The CBP patients have been examined by physicians and met the following criteria: (1) minimum duration of low back pain: for 6 months; (2) pain classified as ‘non-specific low back pain’ (no indication for nerve root problems and radiation to foot or toes, numbness and/or paraesthesia; straight leg raising test caused no leg pain); (3) magnetic resonance imaging (MRI) of the spine only indicated age-related wear and tear but no spinal disorders or disc pathology; (4) no psychiatric disorders, no disease associated to small fiber pathology (e.g., diabetes mellitus), no other chronic disorder; (5) no use of medication (except contraceptive) for at least 48 h prior to the experiment (requested before scanning). All participants were native German speakers and right-handed as assessed by the Edinburgh Handedness Inventory (EHI) [36]. None of the healthy controls reported former subacute or chronic pain episodes (longer than one month), any neurological, psychiatric or other chronic disorder. Because depression may alter the processing of pain-related words [37], depressive symptoms were assessed with a German version of the Beck Depression Inventory-II (BDI-II) [38]. For the assessment of catastrophizing thoughts and persuasions, all subjects completed the Pain Catastrophizing Scale (PCS, [39]; German version: [40]). In accordance with the Declaration of Helsinki, written informed consent was obtained from each participant before the study, and the Ethics Committee of the Friedrich Schiller University approved the experiment.

### 2.2. Verbal Stimuli

Verbal stimuli included pain-related and non-pain-related negative, neutral, and positive adjectives. In a pilot study, 40 words were selected, and rated for valence, arousal, and pain relevance. Pain-related adjectives, affectively negative adjectives, and positive adjectives were matched for arousal. In addition, pain-related and negative adjectives were also matched for valence. Furthermore, word categories were matched according to the number of syllables and frequency in German language (COSMAS II database, http://www.ids-mannheim.de/cosmas2/). For a more detailed description of stimulus selection and the stimulus set, see Richter, Eck, Straube, Miltner and Weiss [32].

### 2.3. Experimental Procedure

**Figure 1:**
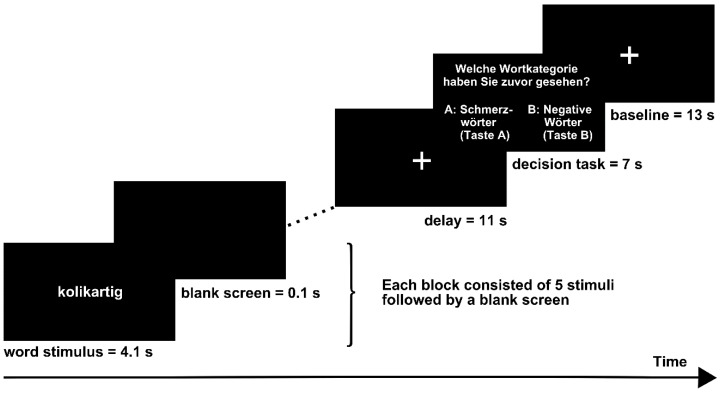
Stimulus protocol.

Examples of each word category were presented while participants were familiarized with the experimental procedure prior to the experiment. A video beamer projected the stimuli onto a screen mounted on the head coil of the MRI scanner. The experimental design is displayed in Figure 1. Subjects were instructed to focus on the semantics of the words by generating a mental image of a situation associated with the word. To increase compliance, subjects were told that they would be asked for examples of their imaginations after the experiment. All subjects were able to associate appropriate mental images to the words. For example, neutral word “traubenförmig” (“aciniform”) was frequently associated with wine grapes and positive word “wärmend” (“warming”) was commonly associated with an oven. Word stimuli were presented in 16 blocks (4 blocks of each word category). Each block consisted of five words (belonging to one word category); each word was displayed for 4.1 s and was followed by a blank screen for 0.1 s. Each block was followed by a delay phase in which a fixation cross was presented for 11 s and a subsequent interval of 7 s. During this interval, subjects were requested to choose the correct word category from two categories presented (e.g., A = pain-related, B = negative). Subjects responded by a MRI-compatible button response box fixed below their right hand. After the selection, a fixation cross was presented for 13 s. Each word was presented twice throughout the experiment. The order of the words within each block and the order of blocks were pseudo-randomized with the restriction that the same word category was not presented twice in succession. The whole fMRI run lasted 14 min.

After the scanning session, participants rated the mean valence, arousal, and pain relevance of each word category on a 10-point numerical rating scale (NRS), with 0 = “negative/no arousal/not relevant”, and 10 = “positive/maximum arousal/highly relevant”. Following the scanning procedure, all subjects also rated their current back pain on a VAS (visual analogue scale, = “no pain”, 10 = “worst pain imaginable”). The pain ratings were obtained at the end of the experiment to avoid any mutual influence between the rating of words and the pain rating. Furthermore, a rating of task difficulty was requested using a VAS (0 = “very easy”, 10 = “very difficult”). During the study, we introduced the vividness of imagination scale [41] measuring emotional imagination, i.e., the ability to create emotional scenarios in mind. Higher ratings are associated with enhanced vividness of imagination.

### 2.4. Analysis of Behavioral Data

All statistical calculations were carried out using IBM SPSS Statistics 19 (IBM, Armonk, NY, USA). Normal distribution of behavioral data was determined by Kolmogorov-Smirnov Test. Levene’s test was applied to assess the equality of variances across the two groups. Variables were statistically analyzed using Student’s *t*- test if they were distributed normally; otherwise, *χ^2^*-tests were applied. Welch’s *t*-test was used for variables with unequal variances across groups. Differences between CBP patients and HC were evaluated for the ratings of arousal, valence, pain relevance, and vividness of imagination of word material [41].

To test differences of word category between patients and HC, separate two-way repeated measurements ANOVAs for mixed experimental design (between-subject factor Group and within-subject factor Word Category) were conducted. We considered values of *p <* 0.05 to be statistically significant.

### 2.5. fMRI-Data Acquisition and Analysis

MRI was obtained by a 3-Tesla magnetic resonance scanner (Tim Trio, Siemens, Medical Systems, Erlangen, Germany). For fMRI, 305 volumes were recorded using a T2* weighted echo-planar sequence (time to echo (TE) = 30 ms, flip angle = 90°, matrix = 64 × 64, field of view (FOV) = 192 mm, scan repeat time (TR) = 2.8 s). Each volume was comprised of 40 axial slices (thickness = 3 mm, no gap, in-plane resolution = 3 × 3 mm) parallel to the intercommissural plane (AC–PC-plane). Additionally, a high-resolution T1-weighted anatomical volume was obtained based on 192 slices with TE = 5 ms, matrix = 256 × 256 mm and resolution = 1 × 1 × 1 mm. Imaging data were pre-processed and analyzed using BrainVoyagerQX, Version 2.8 (Brain Innovation, Maastricht, The Netherlands) and NeuroElf V0.9 (Jochen Weber, SCAN Unit, Columbia University, New York City, NY, USA, http://www.neuroelf.net).

All volumes were realigned to the first volume in order to minimize the effects of head movements on data analysis. Further data pre-processing comprised spatial (6 mm full-width half-maximum isotropic Gaussian kernel) as well as temporal smoothing (high pass filter: 3 cycles per run). Anatomical and functional images were co-registered and normalized to the Talairach space [42]. Statistical analysis of fMRI-data was performed by multiple linear regression of the signal time course at each voxel. The expected blood oxygen level-dependent (BOLD) signal change for each event type (predictor) was modeled by a canonical hemodynamic response function (modified gamma function). A random-effects General Linear Model was used to identify associated brain activity in all acquired slices. To balance between type I and type II errors, we tested whether the detected clusters survived a correction for multiple comparisons. We used the approach as implemented in Brain Voyager which is based on a 3D extension of the randomization procedure described by Forman and colleagues [43,44]. First, voxel-level threshold was set at *p <* 0.05 (uncorrected). Threshold maps were then submitted to a correction for multiple comparisons for each contrast. The correction criterion was based on the estimate of the map’s spatial smoothness and on a Monte Carlo simulation (1000 iterations) for estimating cluster-level false-positive rates. The minimum cluster size threshold yielding a cluster level false-positive rate of 1% was applied to the statistical maps of each contrast [45]. All clusters reported in this article survived this control of multiple comparisons. Main effects were analyzed for the contrast between pain-related words vs. baseline (hypothesis 1; H1). Separate interaction analyses including the factor Group were performed for the relevant contrasts between word categories according to H2: pain-related (weighted 3 times according to the other word categories) vs. all other word categories (negative, neutral, and positive words) and pain-related vs. negative words. For the comparison between CBP patients and HC, the variance of depression (BDI-II) served as covariate in the General Linear Model.

In the next step, we analyzed correlations between VAS pain ratings after the scanning procedure with the relevant differences of parameter estimates (difference: pain vs. negative) for the group of the CBP patients only (HC were excluded because they had no pain, so there is no variance in these parameters allowing a correlative analysis) according to H3. Voxel-level threshold was set at *p <* 0.01 (uncorrected). The map was submitted to a correction for multiple comparisons (see above). After 1000 iterations, the minimum cluster size threshold yielding a cluster level false-positive rate of 1% was applied to the statistical maps.

## 3. Results

### 3.1. Questionnaire and Behavioral Data

*Questionnaire data.* On average, CBP patients reported significantly higher current pain ratings (*M =* 1.69, *SD =* 1.36) than healthy controls (HC) (*M =* 0.04, *SD =* 0.14), *Welch’s t* (12.25) = 4.34, *p <* 0.001 (Table 1). In addition, total BDI-2 scores of CBP patients (*M =* 7.77, *SD =* 5.13) were significantly higher than those of HC (*M =* 2.62, *SD =* 1.76), *Welch’s* *t*(14.78) = 3.424, *p =* .004 (Table 1). According to BDI-scores, only one patient expressed a clinically meaningful depression (score of 20; [38]). Since results remained essentially unchanged after exclusion of this subject, we kept this subject in all further analyses. BDI showed no linear relationship to VAS ratings for current pain (*r*(26) = 0.353, *p =* 0.77). fMRI group differences were calculated with BDI II values as a covariate. There was no significant difference between groups in pain catastrophizing according to the Pain Catastrophizing Scale (PCS; Table 1).

#### H1: Behavioral Effects of Group and Word Category

**Figure 2:**
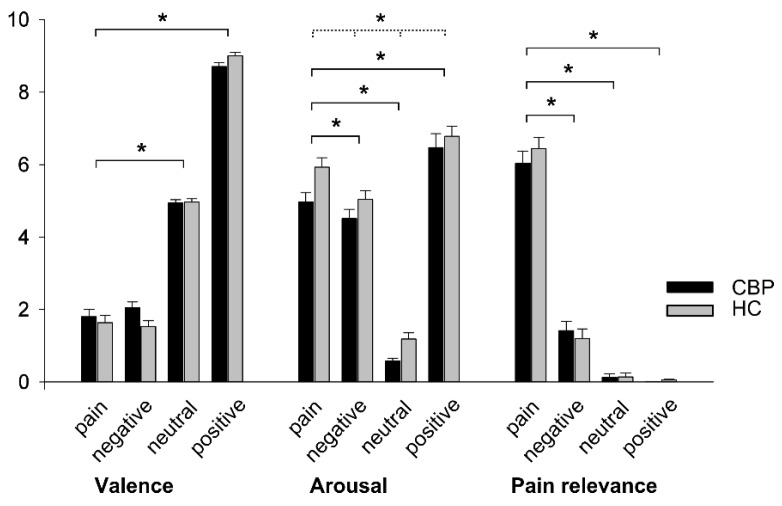
Mean ratings (standard errors) of valence, arousal, and pain relevance of each word category for CBP patients and HC. Valence (0 = “negative”; 10 = “positive”), arousal (0 = “no arousal”; 10 = “maximal arousal”), and pain relevance (0 = “not relevant”; 10 = “highly relevant”). Asterisks (*) indicate significant contrasts of Word Category (black line) and significant main effects of Group (dotted line).

During the experiment, all participants categorized the words properly (M_CB*P*_ = 15.66 and M_HC_ = 15.45 correct out of 16 judgments, see Table 1). ANOVA results of the differences between CBP patients’ and HC subjects’ ratings (regarding post-scanning arousal, valence, and pain relevance) of the word categories are depicted in Figure 2.

*Valence:* Valence of word categories was rated differently as indicated by a significant main effect of Word Category (*F*_3, 54_ = 940.31, *p* < 0.001, *η*^2^ = 0.981). Contrasts were performed by comparing the pain-related word category to the remaining categories: This analysis revealed significant contrasts for neutral vs. pain-related words (*F*_1, 18_ = 315.49, *p <* 0.001, *η*^2^ = 0.946) and positive vs. pain-related words (*F*_1, 18_ = 1803.89, *p <* 0.001, *η*^2^ = 0.990). The contrast negative vs. pain-related words were not significant (*F*_1, 18_ = 0.139, *p =* 0.714, *η*^2^ = 0.008) indicating similar valence of these word categories. No significant main effect of Group (*F*_1, 18_ = 494, *p =* 0.491, *η*^2^ = 0.027) and no significant interaction of Word Category*Group (*F*_3, 54_ = 2.41, *p =* 0.077, *η*^2^ = 0.118) was observed on any valence rating.

*Arousal:* Mauchly’s test indicated that the assumption of sphericity was violated for the main effect of Word Category, (*χ^2^*(5) = 11.66, *p =* 0.040). Therefore, degrees of freedom were corrected using the Greenhouse–Geisser estimate of sphericity (ε = 0.67). As expected, there was a significant main effect of the factor Word Category on arousal ratings (*F*_2.02, 36.35_ = 181.14, *p <* 0.001, *η*^2^ = 0.910), with pain-related words being rated as more arousing than neutral words (*F*_1, 18_ = 505.98, *p <* 0.001, *η*^2^ = 0.966). A significant contrast was observed for the comparison between pain-related and positive words (F_1, 18_ = 13.32, *p =* 0.002, *η*^2^ = 0.425) as well as between pain-related and negative words (F_1, 18_ = 11.87, *p =* 0.003). Likewise, there was a significant main effect of Group on arousal ratings (*F*_1, 18_ = 13.26, *p =* 0.002, *η*^2^ = 0.424) with CBP patients showing lower arousal ratings than HC. No significant interaction Word Category × Group was observed (*F*_2.02, 36.35_ = 0.53, *p =* 0.597, *η*^2^ = 0.028).

*Pain Relevance:* Mauchly’s test indicated that the assumption of sphericity was violated for the main effect of Word Category, (*χ^2^*(5) = 25.57, *p <* 0.001). Therefore, degrees of freedom were corrected using Greenhouse–Geisser estimate of sphericity (ε = 0.65). A repeated measure ANOVA confirmed the effect of Word Category on the rating scores of pain relevance (*F*_1.94, 34.85_ = 413.15, *p <* 0.001, *η*^2^ = 0.958). Contrasts of the factor Word Category confirmed that pain-related words were rated as more pain relevant than negative (*F*_1, 18_ = 487.09, *p <* 0.001, *η*^2^ = 0.947), neutral (*F*_1, 18_ = 763.58, *p <* 0.001, *η*^2^ = 0.977), and positive words (*F*_1, 18_ = 714.04, *p <* 0.001, *η*^2^ = 0.975). There was no significant main effect of Group (*F*_1, 18_ = 0.13, *p =* 0.722, *η*^2^ = 0.007) and no significant interaction between Word Category × Group (*F*_1.94, 34.85_ = 0.83, *p =* 0.440, *η*^2^ = 0.044).

There was also no significant main effect of Vividness of Imagination (*F*_3, 36_ = 1.626, *p =* 0.2, *η*^2^ = 0.119) between word categories and no significant interaction Word Category × Group (*F*_3, 36_ = 0.463, *p =* 0.71, *η*^2^ = 0.037). Average vividness ratings for both groups were 5.58 for neutral words, 5.85 for positive words, 5.65 for negative words and 5.19 for pain-related words. Higher ratings are associated with enhanced vividness of imagination. These data show that both groups were similarly able to generate the requested emotive images associated with the presented words.

### 3.2. Imaging

CBP patients and HC showed activations in a similar network of brain regions in response to viewing pain-related words vs. a fixation cross (baseline). This network includes—among others—the striate and extrastriate cortex of the occipital lobe extending into the fusiform gyrus, widely distributed activations in the frontal lobe bilaterally, bilateral activations of the supplementary motor area (SMA) and pre-SMA, the primary motor cortex (MI) and the anterior cingulate cortex (ACC) (Supplementary Materials Table S1). These activations are in line with previous research [32,34].

#### 3.2.1. H2: Effects of Group and Word Category

**Figure 3:**
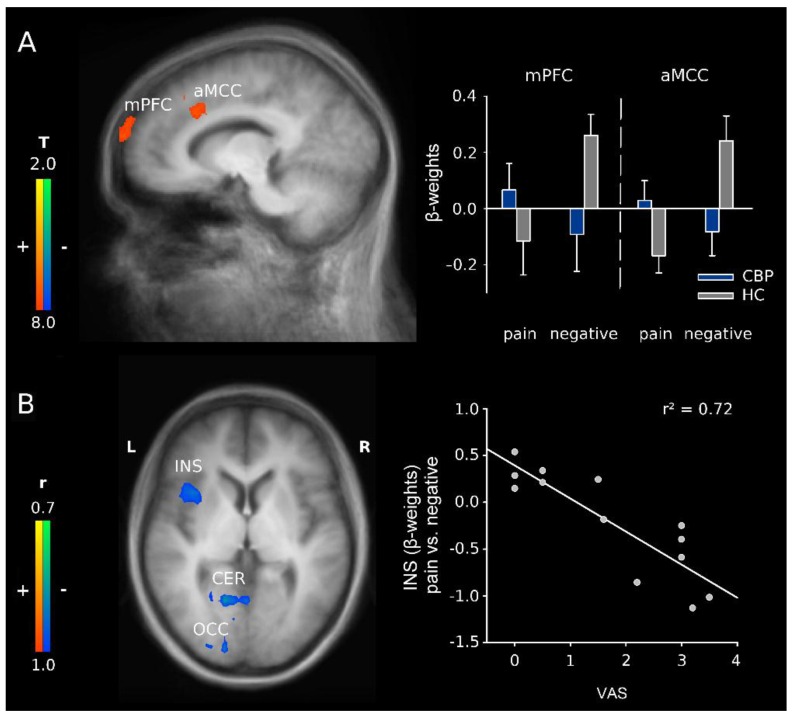
(**A**) activation maps illustrating the interaction between group (CBP patients vs. HC) and word category (pain-related vs. negative adjectives) with activations in the medial prefrontal cortex (mPFC) and anterior midcingular cortex (aMCC) including the dorsolateral prefrontal cortex (DLPFC); x = −10. Right: schematic overview of the β-weights for the aforementioned structures; mean + Standard Error; and (**B**) correlation of current pain (VAS) with the differences in parameter estimates for the contrast pain-related vs. negative adjectives in CBP patients in insula (INS), cerebellum (CER) and occipital cortex (OCC); z = 4. Activations are superimposed on a Talairach template (average of all subjects). Right: correlation plot for the relation of current pain (VAS) and differences in parameter estimates for the contrast pain-related vs. negative adjectives for the anterior insula.

The interaction between Group and Word category (pain-related vs. negative words) revealed increased activations in CBP patients in the medial prefrontal cortex (mPFC), the anterior midcingulate cortex (aMCC), and in the dorsolateral prefrontal cortex (DLPFC; Figure 3A and Table 2). These results are in line with H2. For separate main effects of word category (pain-related words versus negative words), see Supplementary Materials Tables S3 and S4.

We also tested the interaction between Group and Word category for pain-related words and all other word categories [32,34]. This comparison revealed activations in the subgenual anterior cingulate cortex (sACC), the MCC, the posterior cingulate cortex (PCC), bilaterally in the posterior insula, the primary somatosensory cortex (SI) and MI, the fusiform gyrus, the posterior parietal cortex, the mPFC, and in the limbic parahippocampal gyrus (Supplementary Materials Table S2). These results confirm H2.

#### 3.2.2. H3: Correlation Analyses of Word Category in CBP Patients

To test whether the current pain affects the processing of adjectives (H3), a correlation analysis between ratings (VAS) of current pain and the differences in activation between pain-related vs. negative words was performed for the group of CBP patients. We found clusters of negatively correlated activity in several regions including MI and the anterior insula (Figure 3B and Table 3).

## 4. Discussion

The present fMRI study revealed several important results. Firstly, our results support previous findings [32,34] that showed an increase of activation during the processing of pain-related words in several regions of the brain including parts of brain areas that also become activated when exposed to painful stimuli. Secondly, patients suffering from CBP showed stronger activations than HC for pain-related vs. other word categories in several brain structures including the insula and parts of the cingulate cortex. Thirdly, data revealed linear relationships between patients’ current pain and brain activations in CBP patients in a variety of brain structures that are known to be involved in the processing of pain. Behavioral data showed only one effect including factor Group, i.e., a main effect of Group on arousal.

### 4.1. H1: Behavioral Effects of Group and Word Category

Behavioral data show the only effect including factor Group as main effect or interaction for arousal evoked by the different word categories. This effect results from lower arousal ratings in CBP patients compared to HC. There are several reasons that might account for this effect as well as for the absence of significant differences with respect to valence and pain relevance. First, in our sample, CBP patients had relatively low chronic back pain. We discuss this point extensively in Section 4.4 (Study Limitations). Second, ratings of CBP were especially low when the experiment took place. This has previously been reported and might result from distraction and excitement along with the scanning procedure [2,46]. Third, these partly unexpected behavioral results might be due to the permanent exposure to CBP and CBP-related stimuli that the patients suffer from. As a result, habituation to pain-related stimuli may have taken place. Fifth, we also have to take into account that pain-related words were well matched with the other word categories, but they were not specific for CBP; instead, they were referred to pain in general. This might have lowered the impact on our patients specifically suffering from CBP. Nevertheless, there were clear main effects of factor Word Category demonstrating that the expected valence and arousal were specific for each of the word categories. This might serve as a manipulation check of the stimulus material. Moreover, as there were no interaction effects of Word Category × Group on valence, arousal, or pain relevance, these effects could not account for the fMRI results.

### 4.2. H2: Effects of Group and Word Category

CBP patients showed stronger activations than HC during the processing of pain-related words vs. negative words in the medial prefrontal cortex and in a cluster including the aMCC and the DLPFC. In comparison, HC showed stronger brain activation during the processing of negative words and lower brain activation to pain-related words in these structures. Thus, a general difference in processing of pain-related verbal material was observed in CBP patients. Frontal lobe activity during the experience of pain was regularly observed and is generally linked to attention and cognitive processes [47,48]. The mPFC was found to be activated whenever contextual information was used to guide behavior [49]. This structure additionally exerts top-down pain modulation when cognitively demanding tasks interfere with the pain sensation [12,50,51,52]. Thus, activity in the mPFC seems to be strongly modulated during expectation of painful events [12]. In the sense of a priming mechanism, the pain-associated adjectives might have pre-activated a network of structures that were associated with the neuromatrix of pain in the past as a larger neural network. Thus, the cluster including the face area of M1 might be pre-activated due to painful facial expressions [53,54] according to current pain. In a broader sense, similar priming effects have also been shown for action verbs and activation of the sensorimotor cortex [55,56,57]. In the sense of such a priming mechanism and as the mPFC is involved in the recall of recent and remote memory traces [58], we suggest that the processing of painful words is associated with pain-related memories in CBP patients. The DLPFC is known to mediate the cognitive dimension of pain [18,59,60,61,62]. In previous studies, a stronger activation of the DLPFC was found in response to pain-related words as well [32,34]. In the present study, the activation in DLPFC is stronger in CBP patients than in HC, presumably, because these patients are prone to perceive pain more frequently and more seriously than HC. Activity of the aMCC has repeatedly been found for the subjective experience of pain [63]. More specifically, the aMCC seems to integrate pain processing and motor function [64,65]. Thus, structures that are commonly activated when CBP patients are exposed to current painful stimuli seem to be equally activated by words that indicate or connote pain.

In line with previous findings [32], the interaction between pain-related and other word categories revealed enhanced activation in several brain regions including sACC, the aMCC, the ventral posterior cingulate gyrus, the fusiform gyrus, the parahippocampal gyrus, and the posterior insula. Most of these brain areas are implicated in the experience of pain. For example, sACC is involved in the processing of emotional aspects of pain [66], but also in the processing of anxiety and stress [67]. The stronger activation of this structure in CBP patients suggests that pain-related words bear an elevated level of emotional salience and increased stress relatively to non-pain-related words of negative valence for CBP sufferers. It thus might be that their attention is more frequently focused on potential painful threats in the environment.

### 4.3. H3: Relationship to Current Pain

The correlation between current pain (VAS) and differences in activation between pain-related vs. negative words revealed negative correlations in MI and the anterior insula. This is in contrast to our hypothesis H3, i.e., we only found structures where the difference in activation between pain-related and negative words correlated negatively with VAS, but no structure with a positive linear correlation. This result might be due to several reasons. One possible explanation might be that current pain results in a constant activation of these structures. With fMRI, it is not possible to demonstrate this activation as the statistics belong to differences. However, if a pre-activation exists, then the activation of these structures by pain-related words might become less efficient due to pre-activation. Thus, the anterior insula might be pre-activated as a result of current pain due to its significance for the processing of salience information [17,68], which is a core feature of pain [15]. An alternative interpretation might result from the pain-inhibiting pain effect [69,70]. It is well known that two pain stimuli interact by different mechanisms influencing each other [52]. In this sense, chronic pain might result in a lower activation to a pain-related stimulus, even when this stimulus is a pain-related word.

### 4.4. Study Limitations

One limitation of the present study is its relative small number of participants. Strict inclusion criteria and exact matching of participants’ gender and age in both groups made recruitment difficult. However, even in this rather small sample of 13 subjects per group, we revealed significant differences in fMRI activations. Another important limitation of our study is that the CBP patients showed a comparatively low CBP intensity and a relatively low tendency to pain catastrophizing. These characteristics of our CBP patients might result from our inclusion criteria that were already at the advertisement, namely the request not to use any medication (beside contraceptive) 48 h prior to the experiment. This might have deterred more seriously affected CBP patients from participating in our experiment. This limitation is not only important with respect to explaining part of the behavioral results, but it should also be taken into account for the generalizability of the fMRI results. Nevertheless, the magnitudes of pain intensity ratings differed highly significantly from HC. In addition, CBP patients were significantly more depressed than HC, indicating an impairment of everyday life due to chronic pain states. Therefore, in the sense of generalizability, we would expect that our results rather underestimate the effect of CBP. Another limitation is that fMRI group differences were calculated using depression (BDI values) as covariates. Furthermore, our subjects were requested to attend to the presented words and to produce images in their mind with respect to these words. However, we were not able to control whether subjects fulfilled the requests. Future research might investigate whether the present results will remain stable when subjects do not attend or are not requested to imagine related scenes, as well as when CBP patients are more affected than those patients of our sample.

## 5. Conclusions

In summary, the present results revealed that CBP patients compared to HC show enhanced activations to pain-related words in brain structures commonly activated while processing painful events and while processing words with strong associations to pain. Thus, processing of verbal pain-related information is emphasized and particularly meaningful for chronic pain sufferers. However, as differences in brain activations to verbal expressions of pain vs. negative words became smaller, the stronger the current pain was in the CBP patients. These results are in accordance with the associative network theory [56], indicating a significant and systematic interplay between word and pain processing that is enhanced during chronic pain states.

## Figures and Tables

**Table 1:** Demographic and clinical characteristics as well as behavioral data of chronic back pain patients (CBP) and healthy controls (HC).

	CBP	HC			
***Sex***					
**Male/Female**	2/11	2/11			
***Age* (in years)**	44.31 ± 12.15	46.46 ± 10.19			
**Range**	23–56	24–58			
***Pain history***					
**6–12 months**	*N* = 2	*N* = 0			
**2–5 years**	*N* = 4	*N* = 0			
**>5 years**	*N* = 7	*N* = 0			
***Pain intensity***			*t*	*df*	*p*
**Mean pain intensity (VAS ^a^ recent 4 weeks)**	3.31 ± 1.83	0.09 ± 0.30	5.72	10.53	<0.001
**Strongest pain (VAS recent 4 weeks)**	5.14 ± 1.85	0.27 ± 0.90	6.27	12.76	<0.001
**Current pain (VAS post scanning)**	1.72 ± 1.34	0.05 ± 0.15	4.15	10.25	0.002
***BDI*^b^*score***	7.77 ± 5.13	2.62 ± 1.76	3.50	12.12	0.004
***Pain Catastrophizing Scale (PCS)***	14.08 ± 6.11	11.82 ± 7.04	0.61	24	0.550
**Rumination**	5.46 ± 3.46	5.09 ± 3.27	0.12	24	0.905
**Helplessness**	4.85 ± 3.11	4.00 ± 2.61	0.49	24	0.632
**Magnification**	3.77 ± 2.32	2.73 ± 2.19	0.98	24	0.351
***Task difficulty*^c^**	1.38 ± 1.50	1.38 ± 1.50	0	24	1
			*χ^2^*		
***Correct word categorization*^d^**	15.66 ± 0.79	15.45 ± 1.65	0.04	1	0.865

Note: Values are mean ± SD; ^a^ Visual Analogue Scale (VAS): 0 = ‘‘no pain’’, 10 = ‘‘strongest pain imaginable”; ^b^ BDI = Beck Depression Inventory; ^c^ Visual Analogue Scale (VAS): 0 = “very easy”, 10 = “very difficult”; ^d^ correct categorizations out of 16 judgments.

**Table 2:** Activations to pain-related versus negative words in the comparison between CBP patients and HC.

x	y	z	Cluster Size	*t*-Value	Brain Region	Laterality	Brodmann Area
−20	13	35	76	4.92	anterior cingulate cortex/dorsolateral prefrontal cortex	R/L	32
−10	−76	64	54	3.97	medial prefrontal cortex	L	10

Listed are clusters of activation with an uncorrected cluster threshold of *p <* 0.05. Talairach coordinates are provided for the maxima of the respective cluster. The corresponding neuroanatomical regions, the Brodmann areas, and the laterality (L, left; R, right) are described.

**Table 3:** Correlations between current pain ratings (VAS) and the differences in activation between pain-related vs. negative words in CBP patients.

x	y	z	Cluster Size	*r*-Value	Brain Region	Laterality	Brodmann Area
39	13	2	48	−0.83	insula	R	13/44
45	−65	21	56	−0.84	medial temporal cortex	R	39
−16	−62	−11	108	−0.84	cerebellum	L	
5	−87	18	193	−0.88	occipital cortex	R	17/18/23
−43	−15	41	40	−0.90	precentral cortex (MI)	L	4
−4	−77	39	106	−0.91	parietal cortex/occipital cortex	L	19/7
14	−55	−8	211	−0.96	cerebellum	R	19

Listed are clusters of activation with an uncorrected cluster threshold of *p <* 0.01. Talairach coordinates are provided for the maxima of the respective cluster. The corresponding neuroanatomical regions, the Brodmann areas, and the laterality (L, left; R, right) are described.

## Supplementary Materials

The following are available online at www.mdpi.com/2227-9032/4/3/54/s1, Table S1: Activations to pain-related words versus baseline for CBP patients and HC, Table S2: Activations to pain-related versus all other word categories in the comparison between CBP patients and HC, Table S3: Activations to pain-related words versus negative words for HC, Table S4: Activations to pain-related words versus negative words for CBP patients.

## Abbreviations

The following abbreviations are used in this manuscript:

CBP	chronic back pain
HC	healthy control
fMRI	functional magnetic resonance imaging
BOLD	blood oxygen level-dependent
DLPFC	dorsolateral prefrontal cortex
ERP	event-related potential
VAS	visual analogue scale
NRS	numerical rating scale
mPFC	medial prefrontal cortex
INS	insula
CER	cerebellum
OCC	occipital cortex
SMA	supplementary motor area
MI	primary motor cortex
ACC	anterior cingulate cortex
PCC	posterior cingulate cortex
MCC	midcingular cortex
sACC	subgenual anterior cingulate cortex
aMCC	anterior midcingulate cortex
