# Supplementary material for: Enhanced Brain Responses to Pain-Related Words in Chronic Back Pain Patients and Their Modulation by Current Pain
Source: Healthcare (Basel). 2016 Aug 10;4(3):54. doi: 10.3390/healthcare4030054 (PMC5041055; doi:10.3390/healthcare4030054)
Supplement: Supplementary file 1 [file healthcare-04-00054-s001.docx]

Supplementary Materials: Enhanced Brain Responses to Pain-Related Words in Chronic Back Pain Patients and Its Modulation by Current Pain

Alexander Ritter, Marcel Franz, Christian Puta, Caroline Dietrich, Wolfgang H. R. Miltner and Thomas Weiss

**Table S1.** Activations to pain-related words versus baseline for CBP patients and HC.

| **x** | **y** | **z** | **Cluster Size** | ***t*-Value** | **Brain Region** | **Laterality** | **Brodmann Area** |
| --- | --- | --- | --- | --- | --- | --- | --- |
| −35 | −80 | −4 | 921 | 4.44 | inferior occipital cortex/cerebellum | L | 18/19 |
| −50 | 21 | 16 | 1252 | 4.13 | frontal cortex/inferior frontal cortex/supplementary motor area/pre-supplementary motor area/primary motor cortex (MI) | L | 45 |
| 20 | −78 | −17 | 280 | 3.71 | inferior occipital cortex/fusiform cortex/cerebellum | R | 18/19 |
| −4 | 15 | 53 | 153 | 3.67 | superior frontal cortex | R/L | 6/8 |
| −60 | −36 | 4 | 107 | 3.37 | medial temporal cortex | L | 22 |
| 2 | 42 | 15 | 1199 | −3.88 | frontal cortex/medial frontal cortex/anterior cingulate cortex | R/L | 9/10/32 |
| −2 | −64 | 25 | 4646 | −4.04 | parietal cortex/occipital cortex/posterior cingulate cortex | R/L | 31 |

Listed are clusters of activation (3 × 3 × 3 mm³) with an uncorrected cluster threshold of *p* < 0.01. Talairach coordinates are provided for the maxima of the respective cluster. The corresponding neuroanatomical regions, the Brodmann areas, and the laterality (L, left; R, right) are described.

**Table S2.** Activations to pain-related versus all other word categories in the comparison between CBP patients and HC.

| **x** | **y** | **z** | **Cluster Size** | ***t*-Value** | **Brain Region** | **Laterality** | **Brodmann Area** |
| --- | --- | --- | --- | --- | --- | --- | --- |
| 4 | 5 | −12 | 57 | 5.28 | subgenual anterior cingulate, subcallosal gyrus | R | 25/34 |
| −20 | 13 | 35 | 76 | 4.92 | precentral gyrus, postcentral gyrus | L | 3/4/6 |
| 16 | −7 | 54 | 175 | 4.73 | medial frontal gyrus, midcingulate cortex | R | 6/24 |
| −46 | −9 | −13 | 73 | 4.35 | posterior insula, middle temporal gyrus | L | 21 |
| −10 | −69 | 54 | 78 | 4.15 | superior parietal lobule, precuneus | L | 7 |
| 12 | −42 | 3 | 51 | 3.71 | parahippocampal gyrus, ventral posterior cingulate cortex | R | 19/27/29/30 |
| 13 | −76 | 49 | 87 | 3.55 | precuneus | R | 7 |
| 51 | −37 | 17 | 44 | 3.55 | superior temporal gyrus | R | 40/41/42 |
| 31 | −13 | 5 | 80 | 3.51 | posterior insula | R | / |
| −61 | −56 | 6 | 49 | 3.47 | middle temporal gyrus | L | 22 |
| −27 | −38 | −15 | 28 | 3.43 | parahippocampal gyrus | L | 37 |
| −27 | −38 | −15 | 65 | 3.43 | fusiform gyrus | L | 20/37 |

Listed are clusters of activation with an uncorrected cluster threshold of *p* < 0.05. Talairach coordinates are provided for the maxima of the respective cluster. The corresponding neuroanatomical regions, the Brodmann areas, and the laterality (L, left; R, right) are described.

**Table S3.** Activations to pain-related words versus negative words for HC.

| **x** | **y** | **z** | **Cluster Size** | ***t*-Value** | **Brain Region** | **Laterality** | **Brodmann Area** |
| --- | --- | --- | --- | --- | --- | --- | --- |
| −10 | −19 | 31 | 24 | 5.61 | Posterior cingulate cortex | L | 23 |
| 22 | −69 | 6 | 105 | 4.09 | inferior occipital cortex/extrastriate cortex | R | 17/18/19 |
| 16 | −71 | −32 | 50 | 4.12 | cerebellum | R |  |
| −45 | 26 | −1 | 31 | 3.37 | Inferior frontal gyrus/operculum | L | 47 |
| −43 | 13 | −13 | 21 | 6.10 | Superior temporal gyrus/operculum | L | 38 |
| −47 | −43 | −23 | 23 | 3.33 | fusiform gyrus | L | 31 |

Listed are clusters of activation with an uncorrected cluster threshold of *p* < 0.05. Talairach coordinates are provided for the maxima of the respective cluster. The corresponding neuroanatomical regions, the Brodmann areas, and the laterality (L, left; R, right) are described.

**Table S4.** Activations to pain-related words versus negative words for CBP patients.

| **x** | **y** | **z** | **Cluster Size** | ***t*-Value** | **Brain Region** | **Laterality** | **Brodmann Area** |
| --- | --- | --- | --- | --- | --- | --- | --- |
| −38 | −30 | 35 | 25 | 3.84 | premotor cortex | L | 6 |
| −45 | 8 | 26 | 22 | 3.64 | premotor cortex | L | 6 |
| −17 | −72 | 22 | 19 | 3.69 | visual association cortex | L | 18 |
| 45 | 41 | 2 | 160 | 5.61 | dorsolateral prefrontal cortex | R | 46 |
| 4 | 14 | −10 | 78 | 4.36 | subgenual anterior cingulate cortex | R/L | 25 |
| 28 | 1 | −28 | 93 | 3.74 | temporopolar area | R | 38 |
| −27 | 8 | −25 | 31 | 4.00 | temporopolar area | L | 38 |

Listed are clusters of activation with an uncorrected cluster threshold of *p* < 0.05. Talairach coordinates are provided for the maxima of the respective cluster. The corresponding neuroanatomical regions, the Brodmann areas, and the laterality (L, left; R, right) are described.
